# Changes in Self-Reported Concussion History after Administration of a Novel Concussion History Questionnaire in Collegiate Recreational Student-Athletes

**DOI:** 10.3390/sports5040095

**Published:** 2017-12-17

**Authors:** Adam Copp, Monica R. Lininger, Meghan Warren

**Affiliations:** Department of Physical Therapy and Athletic Training, Northern Arizona University, Flagstaff, AZ 86011, USA; ac3329@nau.edu (A.C.); monica.lininger@nau.edu (M.R.L.)

**Keywords:** concussion education, reporting consistency, club sports, intramural sports

## Abstract

Research has shown that exposure to a concussion definition (CD) increases self-reported concussion history (SRCH) immediately, however, no research has been performed that examines the effects of exposure to a CD on SRCH over time. Collegiate recreational student-athletes (RSAs) have limited access to monitoring and supervision by medical staff. As such, recognition of concussion symptoms and need for medical management oftentimes falls upon the RSA. The purpose of this study was to assess the effect of a novel questionnaire on the SRCH of RSAs. A two-part questionnaire was sent to RSAs participating is sports with a greater than average risk of concussion at a university in Arizona. Data from 171 RSAs were analyzed to assess the change in RSAs’ suspected concussion estimates pre- and post-exposure to a CD and concussion symptom worksheet, as well as over the short-term (2.5 months). Approximately one-third of RSAs reported an increase in suspected concussion estimates immediately following exposure to the questionnaire, but the change was not maintained over the short-term. The results suggest that a single exposure to a CD is ineffective at increasing short-term SRCH estimates.

## 1. Introduction

In a given year in the US, approximately 1.6 to 3.8 million concussions befall athletes in competitive sports and recreational activities, with up to half possibly going unreported [1,2]. A substantial proportion are likely unreported because athletes may be unaware of common signs and symptoms of concussion [3,4]. Collegiate recreational student-athletes (RSAs) may be especially at risk for underreporting, as injury monitoring and supervision by medical staff (i.e., physician, certified athletic trainer) are non-standardized for this population [5].

A 2014–2015 data set of 330 colleges and universities compiled by the National Intramural and Recreational Sports Association (NIRSA) reported that the typical collegiate institution offers 20 intramural sports, with 1000 games played annually, as well as an additional 20 sports clubs [6]. High concussion risk sports, like bicycling and rugby, are often unique to club sports programs [7]. Recreational sports teams are often student-run organizations, with limited access to medical coverage [8]. As such, recognition of concussion symptoms and need for medical management oftentimes falls solely upon the RSA.

Self-reported concussion history is a common but inaccurate assessment of lifetime concussion exposure [9]. In an attempt to standardize the assessment of concussion history, the International Conference on Concussion in Sport recommended asking specific questions related to symptoms, and not merely the perceived number of concussions [10]. Following the conference’s recommendation, several studies reported that concussion symptom questionnaires may be more accurate at quantifying concussion history compared to simply asking perceived number of concussions [3,11,12].

More recently, researchers have asked current and former athletes to estimate concussion history pre- and post-exposure to a current definition of concussion [9,13]. Athletes concussion history estimates increased significantly following exposure to the concussion definition [9,13]. However, whether or not providing a definition had a lasting effect on participants’ self-reported concussion history was not assessed.

Can an assessment tool also function as an educational tool? The current study sought to answer this question in an athlete population historically lacking formal injury monitoring and supervision by medical staff. By presenting a current concussion definition in a format that utilized design components from concussion symptom questionnaires, the authors hoped to discover if the process of assessment has short-term effects on concussion history reporting.

As such, the purpose of the current study was to assess the change in the number of suspected concussions immediately after providing a concussion definition and symptom worksheet via an online questionnaire, and then again at 2.5 months after the initial measurement. We hypothesized that RSAs would increase self-reported concussion history estimates immediately, and that the increase would be maintained over the short-term. An exploratory purpose was to examine factors associated with change in concussion reporting.

## 2. Materials and Methods

### 2.1. Participants

RSAs 18 years and older participating in club or intramural sports with a greater than average risk of concussion [14,15,16] at a university in Arizona were invited to participate in the study. The sports included: intramural (IM) basketball, flag football, soccer and volleyball; and club (C) beach volleyball, cycling, equestrian sports, gymnastics, ice hockey, lacrosse, rugby, soccer, volleyball, roller derby, roller hockey, and ski/snowboard. There were 2094 e-mail invitations sent out to RSAs on 8 February 2017. The study was approved by the Northern Arizona University (1006918) Institutional Review Board, and potential participants completed an online informed consent prior to completing the study.

### 2.2. Instrumentation and Procedures

SurveyMonkey (SurveyMonkey Inc., San Mateo, CA, USA) was used to create and administer the initial and follow-up (2.5 months) questionnaires. Emails with links to the questionnaires were sent to RSA email addresses provided by the Campus Recreation Department at Northern Arizona University. Reminder emails were sent using SurveyMonkey to RSAs who had not completed the questionnaire three days following initial and follow-up questionnaire email requests.

The questionnaire collected demographic data (age, sex, primary sport, IM or C, years of participation in primary sport, and other sports currently played). The RSAs were asked to estimate both the number of diagnosed and suspected concussions experienced since 14 years of age. A description and definition of concussion, adapted from Robbins et al., was then provided: “Some people have the misconception that concussions only happen when you black out after a hit to the head or when the symptoms last for a while. But, a concussion has occurred with any blow to the head that caused symptoms for any amount of time” [9] (p. 101). In addition, a concussion symptom worksheet derived from the concussion symptom list from Randolph et al. [17] and the concussion symptom survey from LaBotz et al. [11] was presented. The RSAs were asked to estimate the number of times they had experienced each of the listed concussion symptoms following any blow to the head. After exposure to the concussion definition and completing the concussion symptom worksheet, RSAs were asked again to estimate the number of diagnosed and suspected concussions experienced since 14 years old. See Supplementary Material S1 for the initial questionnaire.

A follow-up questionnaire was sent to all RSAs who completed the initial questionnaire 2.5 months later (30 April 2017). The RSAs were asked if they had been diagnosed with or suspected a new concussion since completing the initial questionnaire in February 2017, and if so, how many times. Following, RSAs were asked again to estimate total diagnosed and suspected concussions experienced since 14 years old. See Supplementary Material S2 for the follow-up questionnaire.

### 2.3. Data Preparation

The concussion estimates provided pre-exposure to the concussion definition were labeled as T0, or baseline. The number of suspected concussions provided immediately following exposure to the concussion definition and symptom worksheet were labeled as T1. The suspected concussions provided at the follow-up questionnaire (2.5 months) were labeled as T2.

Participant data were categorized by change in suspected concussion estimates between assessments. Participants were categorized as ‘Increased’ if there was an increase in suspected concussions between T1 and T0 assessment interval. Participants were categorized as ‘No Change’ if suspected concussion estimates remained the same between the T1 and T0 assessment interval. Participants were categorized as ‘Decreased’ if there was a decrease in suspected concussions between T1 and T0 assessment interval.

For T2 to T1 assessment interval, participants were categorized as ‘No Change’ if suspected concussion estimates remained the same. Participants were categorized as ‘Changed’ if there was an increase or decrease in suspected concussions estimates between T2 and T1 assessment interval.

To account for variability in age and the number of years an RSA participated in the primary sport, years in sport was divided by age to create a binary variable of greater or less than half the RSA’s life as a measure of experience in sport. Primary sport was categorized as soccer/rugby, flag football, volleyball, basketball, and other (e.g., cycling).

Inclusion for participant response data required that T0 and T1 diagnosed and suspected concussion estimates were complete and Yes/No responses were consistent with concussion estimates (example of an inconsistent Yes/No response: “… diagnosed … with a concussion?”: “Yes”; “If … YES … how many times …?”: “0”). One participant’s data was excluded due to outliers in several variables. Participants who reported 0 concussions at T0, T1, and T2 were only assessed for demographic characteristics.

### 2.4. Statistical Analysis

Descriptive statistics were calculated using frequencies or percentages for categorical variables and medians and ranges for continuous variables. Categorized change in suspected concussion estimates was reported for each assessment interval. A histogram was created to display diagnosed plus suspected concussions at T0 (baseline). For the first exploratory analysis (T1 to T0), logistic regression was used due to small cell size in the Decreased group (Table 1), to assess the association between participant characteristics and the categorized change in suspected number of concussions. Nine RSAs in the Decreased group were excluded for this analysis. Sex, years in sport accounting for age, and baseline (T0) concussion sum (diagnosed plus suspected) were the independent variables. Baseline (T0) concussion sum was categorized into 0, 1–2, and 3+ concussions [18]. The dependent variable was the nominally categorized change from T1 to T0 (Increased and No Change).

Of the 171 RSAs that completed the T0 and T1 measurements and reported at least 1 concussion, 109 RSAs also provided complete data at T2. For the second exploratory analysis, a logistic regression model was used to determine the association between the same independent variables stated earlier, and the categorized change (Changed and No Change) from T2 to T1. For all regression analyses, odds ratios (OR) and 95% confidence intervals (CI) were calculated.

Alpha was set to 0.05 for statistical significance testing, and analyses were completed with IBM SPSS Statistics for Windows Version 24 (IBM Corp., Armonk, NY, USA).

## 3. Results

### 3.1. Participants and Descriptive Data

**Figure 1:**
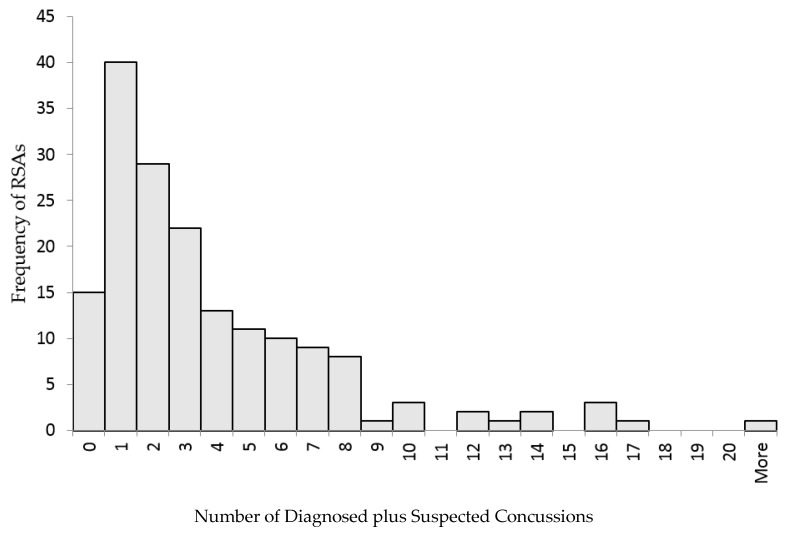
Distribution of diagnosed plus suspected concussions at T0 (baseline) for RSAs (*N* = 171).

From the invitation to participate in the study, 377 RSAs responded to the initial questionnaire, 259 of which had complete questionnaires and met inclusion criteria. Eighty-eight of the 259 RSAs reported no history of concussion; therefore, initial analysis was completed with 171 RSAs. There was no statistical association between those not included in the analysis (*n* = 259) and those included (*n* = 171) on sex (*x*^2^_1_ = 0.24, *p* = 0.62) or age (*U* = 5435, *p* = 0.18). Per the campus recreation department at the university where the data were collected, approximately 30% of the participants in club and intramural sports are female, and approximately 40% were included in the analysis. At the 2.5 month follow-up questionnaire, 109/171 (63.7%) RSAs responded. The median age of the sample was 20 years, and about 40% of the respondents were female (Table 2). The highest number by sport (56/171, 33%) participated in rugby or soccer, with the fewest (17/171, 10%) involved in basketball. On average, the RSAs reported participation in their primary sport for 10 years. As seen in Figure 1, most of the RSAs had one diagnosed plus suspected concussion at T0 in the positively skewed distribution. No RSAs were diagnosed with a concussion between T1 and T2.

### 3.2. Main Results

Immediately following exposure to the concussion definition and concussion symptom worksheet, 52/171 RSAs (30.4%) increased suspected concussion estimates (Increased), 110/171 RSAs (64.3%) reported no change (No Change), and 9/171 RSAs (5.3%) decreased suspected concussion estimates (Decreased).

At the follow-up questionnaire (2.5 months), 41/109 RSAs (37.6%) reported no change in suspected concussion estimates (No Change), and 68/109 RSAs (62.4%) reported increased or decreased suspected concussion estimates (Changed). Of these RSAs, 21 reported increased and 47 reported decreased suspected concussion estimates.

Of the RSAs who completed both initial and follow-up questionnaires, 36/109 (33%) increased suspected concussion estimates immediately following exposure to the concussion definition and concussion symptom worksheet. Of those 36 RSAs, 4 (11.1%) reported no change (No Change) and 32 (88.9%) increased or decreased suspected concussion estimates (Changed) at the follow-up questionnaire (2.5 months). Seventy of the 109 RSAs reported no change in suspected concussion estimates (No Change) immediately following exposure to the concussion definition and concussion symptom worksheet. Of those 70 RSAs, 36 (51.4%) reported no change (No Change) and 34 (48.6%) increased or decreased suspected concussion estimates (Changed) at the follow-up questionnaire. Three of the 109 RSAs reported decreased suspected concussion estimates (Decreased) immediately following exposure to the concussion definition and symptom worksheet. Of those three RSAs, one reported no change in suspected concussion estimates (No Change) and two reported increased or decreased concussion estimates (Changed) at the follow-up questionnaire.

There was no association of sex (Odds ratio [OR] = 1.43 (95% confidence interval [CI] 0.67–3.07) or experience (OR = 0.56 (0.26–1.20)) with categorized change in suspected concussions from T1 to T0, with Increased as the referent group. However, as seen in Table 3, those RSAs that had 1–2 diagnosed plus suspected concussions at baseline, had statistically greater odds of being categorized as No Change than those with no concussions at baseline. This was also found when comparing those that had three or more concussions at baseline compared to those with no concussions. From T2 to T1, there were no associations of the participants’ demographics with categorized change (Table 4, Changed or No Change), specifically sex (OR = 0.62 (0.23–1.43)) and experience (OR = 0.73 (0.31–1.70)).

## 4. Discussion

The purpose of the study was to assess the immediate and short-term changes in suspected concussions after providing a concussion definition and symptom worksheet to RSAs via an online questionnaire. A secondary purpose was to examine factors associated with change in concussion reporting. Approximately one-third of RSAs reported an increase in the number of suspected concussions immediately following exposure, but the change was not maintained over the short-term (2.5 months). The results suggest that a single exposure to a concussion definition and symptom worksheet is ineffective at increasing short-term self-reported concussion history estimates. Additionally, those with more concussions (diagnosed and suspected) had lower odds of reporting a change in the number of concussions after completing the concussion symptom worksheet.

The National Collegiate Athletic Association (NCAA) mandates that participating institutions provide concussion education materials for varsity student-athletes, however, the content and format are not standardized [19]. Importantly, the recreational student-athletes included in this research exist outside of that mandate [7]. Kroshus et al. [19] assessed the effectiveness of concussion education materials for a group of NCAA male ice hockey teams. The authors discovered that content and delivery varied, and there was no change in knowledge or attitudes post exposure; the educational tools provided to collegiate student-athletes were ineffective [19]. Provvidenza et al. [20] described the importance of developing and evaluating education strategies for effective knowledge transfer concerning concussion education; this was one intention of the present research.

Providing a definition of concussion prior to assessing self-reported concussion history has been shown to increase self-reported concussion history [9,13]. However, previous studies have only assessed immediate changes in concussion reporting [9,13]. The current study assessed suspected number of concussions both immediately and if the information was sustained over time. In agreement with others [9,13], the current study found an immediate effect in number of suspected concussions, but not over time.

The present research was performed with currently playing female and male collegiate RSAs, who have likely been exposed to a more contemporary concussion definition than the predominantly male, former football athlete group studied by Robbins et al. [9]. Seventy-three percent of their study participants increased concussion history estimates after exposure to the concussion definition compared to 30% in the current study. This relatively high percentage is likely related to the age-related differences in concussion education of their sample group, as the mean age of athletes in their study was 44 years.

This study assessed the effectiveness of a different format of concussion definition. Rather than simply presenting a list of common concussion symptoms, participants completed a symptom worksheet, similar to a concussion symptom questionnaire, in which they were asked to estimate the number of times they experienced each symptom. The intention was to require a more thorough self-assessment to encourage short-term carryover of any changes to a participant’s self-reported concussion history. Previous research has documented that concussion symptom questionnaires are more sensitive for assessing concussion histories than simply asking perceived number of concussions [3,11,12]. Athletes increased self-reporting of suspected concussion history immediately following exposure. Whether the increase was due to the symptom worksheet specifically or exposure to a concussion definition in general cannot be discerned in the current study. Of greater importance is the fact that our design and methods were ineffective for short-term concussion knowledge transfer.

There are several limitations to this study. First, those who have experienced more concussions and those who know less about concussions may be more likely to participate. The sample may have been biased towards female participants, as 40% of study participants were female, whereas females only account for 30% of club and IM sport participants. The current categorization of change in suspected concussion estimates may not have appropriately represented how the participant reported. Concussion estimate increases may have been indicative of inconsistent reporting rather than increased awareness of concussion experience. It may have been difficult for some participants to correctly identify concussion experience within our guidelines (i.e., “since turning 14 years old (generally, first year of high school)”). However, as the median age of the participants was 20, recall was possibly less of a concern. Although the definition of concussion used in this study was based on US and international consensus statements, it is important to note that body impacts can result in concussions as well. Almost one-third (32.7%) of participants did not complete the follow-up questionnaire at T2 despite a reminder email. Finally, caution is warranted with the wide confidence intervals with all inferential statistics. Although estimates are not very precise, this is the first study that included only recreational student-athletes, and is an important first step in quantifying concussion prevalence and knowledge in this at-risk population.

Future research should examine other formats for presenting a concussion definition. Specifically, future research should assess the possibility of a dose–response learning effect with exposure to concussion history assessment tools. In addition, future research could ascertain methods to improve response rate to allow for statistical analysis over time without relying too heavily on various imputation methods. Most importantly, it is imperative that future research continue to address the development and assessment of novel educational tools for concussion knowledge transfer. Recreational student-athletes as well as varsity student-athletes should be used as research participants, and both groups should be exposed to concussion education tools when effective ones are discovered. Policy for recreational sports (e.g., mandated concussion education or medical screening prior to participation) should be explored empirically to better understand if this will help minimize the risk of concussion injury. At Northern Arizona University [21], for example, participation in club sports requires a waiver including physician name and contact information, brief medical history questions, allergies, medications, height, and weight. Participation in intramural sports is voluntary and should be undertaken within a person’s own physical and mental health. Participants are encouraged to have a medical examination before participation, but nothing is required. Any mandated changes based on the current study would obviously have far-reaching consequences at the University, including staff and infrastructure. Further research is needed to determine how to best translate the information in the current study into practice.

This study supported prior research that demonstrated athletes’ self-reported concussion history increases immediately following exposure to a concussion definition. Exposure to the initial questionnaire, which included a concussion definition and symptom worksheet, increased suspected concussion estimates in approximately one-third of the sample immediately following exposure. Increases in self-reported concussion history, however, were not maintained over the short-term (2.5 months). The results suggest that a single exposure to a concussion definition is ineffective at increasing short-term self-reported concussion history estimates.

## Figures and Tables

**Table 1:** Frequencies of categorized change for assessment intervals and measure of diagnosed and suspected concussions at T0.

Assessment Interval	Category of Change	Diagnosed plus Suspected at T0			Total
		None	1–2	3+	
T1 to T0 (*n* = 171)	Decreased	0	1	8	9
	Increased	14	11	27	52
	No change	1	57	52	110
Total		15	69	87	171
T2 to T1 (*n* = 109)	Changed	8	19	41	68
	No change	1	23	17	41
Total		9	42	58	109

**Table 2:** Demographic characteristics.

Variables		Total Respondents (*n* = 259)	Total Analyzed (*n* = 171)
Age, Median (Range)		20 (18–30)	20 (18–29)
Female, *n* (%)		106 (40.9%)	68 (39.8%)
Sport, *n* (%)	Rugby/soccer	86 (33.2%)	56 (32.7%)
	Flag football	48 (18.5%)	30 (17.5%)
	Volleyball	20 (7.7%)	18 (10.5%)
	Basketball	36 (13.9%)	17 (9.9%)
	Other	69 (26.7%)	50 (29.2%)
Years in Sport, Median (Range)		10 (0–25)	10 (0–21)
Number of Diagnosed Concussions at T0, Median (Range)		0 (0–13)	1 (0–13)
Number of Suspected Concussions at T0, Median (Range)		0 (0–16)	1 (0–16)

**Table 3:** Number of concussions (percent) for RSAs categorized as Increased versus No Change between T1 to T0 (*n* = 162).

Diagnosed plus Suspected at Baseline	Increased	No Change
None	14 (93.3%)	1 (6.7%)
1–2	11 (16.2%)	57 (83.8%)
3+	27 (34.2%)	52 (65.8%)
Total	53 (32.7%)	109 (67.3%)
Adjusted odds ratio (95% confidence interval) with None as the referent group: 1–2 OR for Increased = 0.01 (0.0001–0.10) 3+ OR for Increased = 0.03 (0.004–0.25)		

**Table 4:** Number of concussions (percent) for RSAs categorized as Changed versus No Change between T2 to T1 (*n* = 109).

Diagnosed plus Suspected at Baseline	Changed	No Change
None	8 (88.9%)	1 (11.1%)
1–2	19 (45.2%)	23 (54.8%)
3+	41 (70.7%)	17 (29.3%)
Total	68 (62.4%)	41 (37.6%)
Adjusted odds ratio (95% confidence interval) with None as the referent group: 1–2 OR for Changed = 8.69 (0.99–76.50) 3+ OR for Changed = 2.71 (0.31–23.86)		

## Supplementary Materials

The following are available online at www.mdpi.com/2075-4663/5/4/95/s1, S1: Initial questionnaire, S2: Follow-up questionnaire.
